# Easy-to-Build and Reusable Microfluidic Device for the Dynamic Culture of Human Bronchial Cystic Fibrosis Epithelia

**DOI:** 10.1021/acsbiomaterials.2c01460

**Published:** 2023-04-05

**Authors:** Claudia Mazio, Laura S. Scognamiglio, Roberta Passariello, Valeria Panzetta, Costantino Casale, Francesco Urciuolo, Luis J. V. Galietta, Giorgia Imparato, Paolo A. Netti

**Affiliations:** † Istituto Italiano di Tecnologia (IIT)—Center for Advanced Biomaterials for Healthcare, Largo Barsanti e Matteucci 53, 80125 Napoli, Italy; ‡ Department of Chemical, Materials and Industrial Production Engineering (DICMAPI), University of Naples Federico II, P.le Tecchio 80, 80125 Naples, Italy; § Interdisciplinary Research Centre on Biomaterials (CRIB), University of Napoli Federico II, P.le Tecchio 80, 80125 Napoli, Italy; ∥ Telethon Institute of Genetics and Medicine (TIGEM), Via Campi Flegrei 34, 80078 Pozzuoli (NA), Italy

**Keywords:** microfluidic chip, in vitro dynamic culture, air−liquid interface, human bronchial epithelium, cystic fibrosis

## Abstract

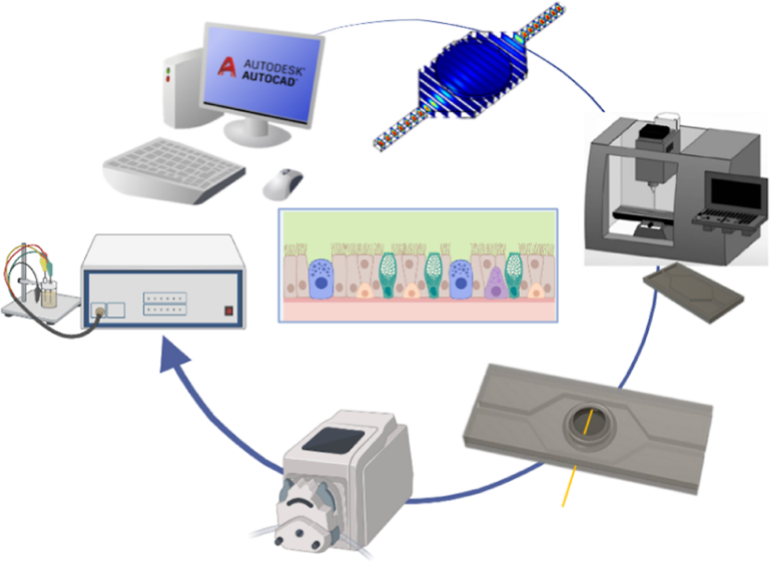

Cystic fibrosis (CF) is one of the most frequent genetic diseases, caused by dysfunction of the CF transmembrane conductance regulator (CFTR) chloride channel. CF particularly affects the epithelium of the respiratory system. Therapies aim at rescuing CFTR defects in the epithelium, but CF genetic heterogeneity hinders the finding of a single and generally effective treatment. Therefore, in vitro models have been developed to study CF and guide patient therapy. Here, we show a CF model on-chip by coupling the feasibility of the human bronchial epithelium differentiated in vitro at the air–liquid interface and the innovation of microfluidics. We demonstrate that the dynamic flow enhanced cilia distribution and increased mucus quantity, thus promoting tissue differentiation in a short time. The microfluidic devices highlighted differences between CF and non-CF epithelia, as shown by electrophysiological measures, mucus quantity, viscosity, and the analysis of ciliary beat frequency. The described model on-chip may be a handy instrument for studying CF and setting up therapies. As a proof of principle, we administrated the corrector VX-809 on-chip and observed a decrease in mucus thickness and viscosity.

## Introduction

Cystic fibrosis (CF) is the most common autosomal recessive disorder in the Caucasian population, with a frequency of occurrence of about 1/3500 newborns.^1^ The disease is caused by mutations in the CF transmembrane conductance regulator (CFTR) gene. The CFTR protein is a chloride channel located at the apical surface of epithelial cells in different organs where it plays a fundamental role in trans-epithelial fluid homeostasis.^2^ Several gene mutations have been identified over the years, mainly reducing the channel number or function. The most common CFTR mutation is the deletion of phenylalanine at position 508 (F508del) which is associated with defective protein folding, resulting in proteasomal degradation and very little or no CFTR reaching the apical membrane of the epithelium.

CFTR dysfunction affects many organs, but lung disease is responsible for most of the severe complications in patients with CF. Indeed, CF patients display a chronic respiratory disease featuring mucus obstruction and bacterial infection.^3^ Furthermore, chronic tissue damage and inflammation may induce airway remodeling and bronchiectasis, which represent severe complications of the pathology.^4,5^ Recent therapies for CF aim to rescue CFTR function. Indeed, CFTR potentiators improve channel opening and ion transit, while correctors enhance CFTR folding and functional insertion into the cell membrane. Thereby, patient therapy depends on CFTR-specific mutation, thus making CF a pathology suitable for personalized medicine.^6^ Animal models fail in reproducing important phenotypic manifestations of human CF disease, imposing limitations on translational research. In this context, the development of human CF models in vitro meets the need for a robust and reliable platform for drug discovery.^7^ Now, primary human bronchial epithelial cells represent the gold standard model for studying CFTR dysfunction and recovery in the lungs. These cells, seeded at a high density on porous inserts, can differentiate at the air–liquid interface (ALI), generating a tight epithelium with cilia and mucus on the apical surface. This is possible, thanks to the presence of basal cells in culture that require about 21–28 days to develop the differentiated tissue.^8^

Recently, lung-on-chip models have been developed to reproduce physio-pathological conditions of the airway and drug testing, to overcome the limits of animal models, and to increase human translatability.^7,9,10^ Indeed, advances in microfluidics allow the controlled spatiotemporal release of drugs. Substances can be administrated from the apical or basolateral side of the sample, by using an aerosol system or the fluidic flow. At the same time, the possibility to integrate multi-organs on the chip enables the spatial organization and continuous communication between different organs, all involved in drug metabolism and function.^10−12^ Several papers have previously demonstrated the advantages of microfluidics in mimicking the dynamic in vivo environment, stimulating tissue differentiation, analyzing the effect of external substances, and studying tissue communication.^13−16^ Huh et al. developed a functional model of the alveolar-capillary interface replicating the mechanism of human breathing on a chip.^9^ Benam et al. reproduced the lung physiology and inflammation model with a differentiated bronchial epithelium and an underlying microvascular endothelium into a microfluidic device composed of an apical and a basal channel separated by a porous membrane in polydimethylsiloxane (PDMS).^17^ By using the same microfluidic system, Plebani et al. mimicked bronchial infection, inflammation, and immune cell recruitment in CF. To the best of our knowledge, this is the first and only CF model of the airway on a chip.^18^ In spite of the straightforward validity of all these previous models, they often suffer from excessive complexity in fabrication. Furthermore, the rationale for the use of fluidic condition was poorly explained, and the microfluidic chip implementation results complex for not specifically equipped research labs.

Here, we developed an easy-to-build and reusable microfluidic chip able to simply host commercial porous inserts such as Transwell/Snapwell of multiple manufacturers and sizes. Other groups already proposed the combination of microfluidics with commercial inserts for ALI culture, but, given the geometry of their systems, they can hold just a type of insert with fixed dimensions. For example, Morgan et al. developed a microfluidic system housing conventional Transwell. They demonstrated that such a device allowed the analysis of the effect of environmental agents on the airway epithelium with higher sensitivity compared to static models.^19^ More recently, the group of Tung demonstrated a device compatible with Transwell inserts and capable of vapor exposure and time-lapse monitoring of alveolar epithelium barrier function.^20^ However, both research’s groups did not explore the possibility to use the device for replicating airway disease, such as CF. The chip we fabricated was transparent and reusable because the epithelial tissue was grown into the removable porous insert and therefore could be separated from the chip after culture, thus allowing for subsequent reuse. The geometry of the chip was based on previous evidence from the literature,^14,21^ but it was adjusted to optimize fluidic simulations in terms of tissue oxygenation, homogeneous shear stress distribution, and differentiation of the CF human bronchial epithelium (HBE-CF). In this work, we compared the morphological and functional features of HBE-CF cultured on-chip (in dynamic) or in a multiwell (static). Afterward, we highlighted the differences between HBE-CF and HBE-NonCF on-chip. Our results demonstrate that the dynamic condition stimulates tissue differentiation with a peak of transepithelial electrical resistance (TEER) at day 3 and mucociliary differentiation in 2 weeks. A discrepancy in electrophysiology (TEER and capacitance), mucus quantity, and viscosity was observed by comparing HBE-CF and HBE-NonCF on-chip. The treatment of the CF epithelium on-chip with the corrector VX809 induced a decrease in mucus viscosity and thickness.

## Materials and Methods

### Cell Source

CF human bronchial epithelial cells carrying F508del mutation (HBE-CF: BE115 and BE86) were provided by the Primary Cell Culture Facility of the FFC (Istituto G. Gaslini, Genova), as well as non-CF human bronchial epithelial cells (HBE-NonCF: BE122 and BE37). Cells were seeded in flasks pre-coated with 10 μg/mL rat tail collagen (for 1 h into the incubator) and expanded using the LHC9/RPM1640 serum-free medium, as previously described, until P3/4.^22,23^ Rat tail collagen and LHC9/RPM1640 were supplied by the Primary Cell Culture Facility of the FFC. Afterward, about 250,000 cells (HBE-CF or HBE-NonCF) were seeded on porous inserts (Corning CLS3470) pre-coated with 10 μg/mL rat tail collagen (for 1 h into the incubator). Cells on porous inserts were cultured in the static condition in multiwell or dynamic culture on-chip. Both in static and dynamic, the epithelia were differentiated at the ALI.

### Design and Fabrication of a Microfluidic Device

**Figure 1:**
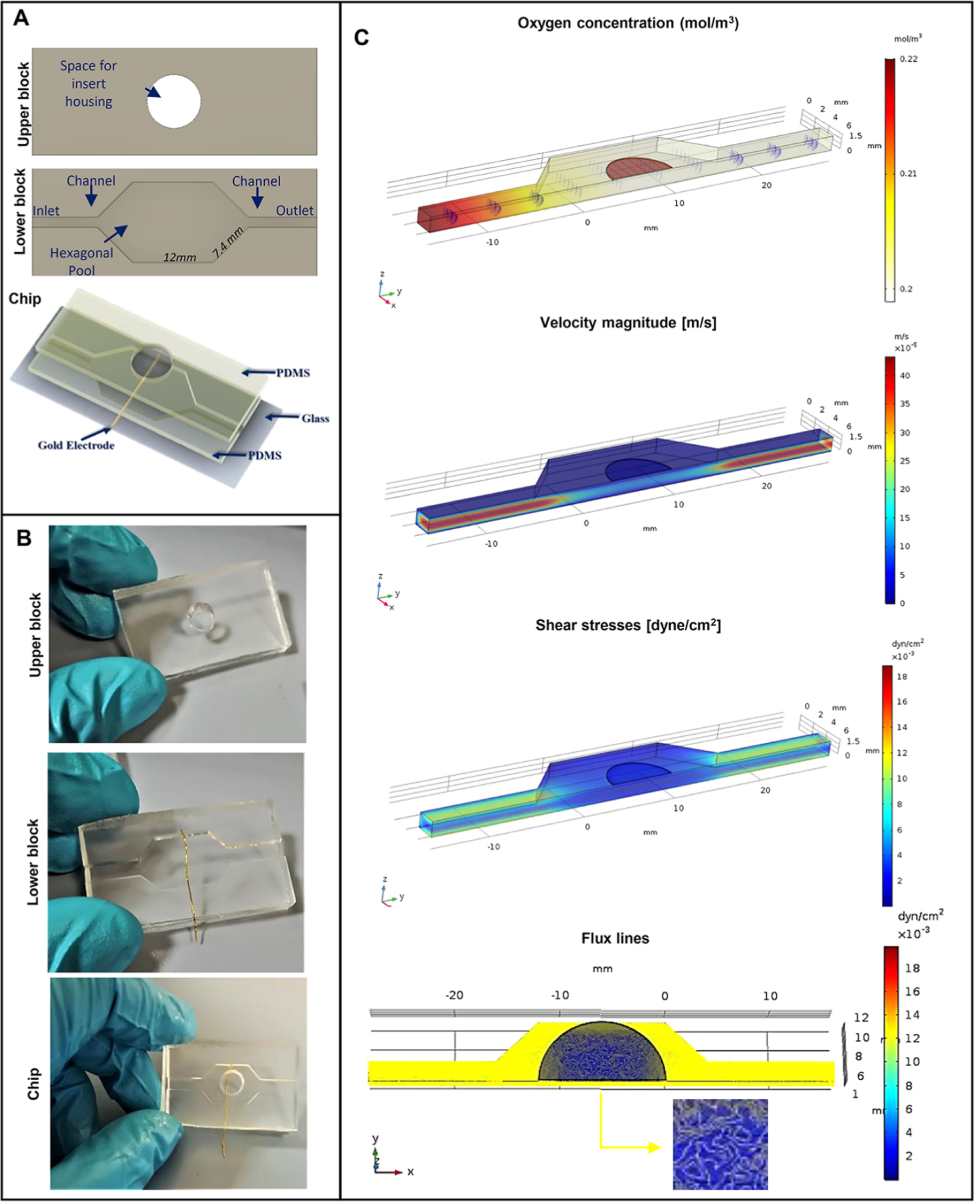
Chip fabrication and simulation of fluidic conditions: (A) design of the microfluidic chip with the top block with the space for Transwell housing and the bottom one composed of the fluidic channel and a central hexagonal pool. A gold electrode was integrated into the device, and the chip was mounted on a glass slide for stabilization; (B) pictures of the top and bottom blocks of the device and the bonded chip; (C) COMSOL simulation of oxygen concentration, flow velocity, shear stress, and flux lines with the related bar of the colormap. The magnification highlights the presence of homogeneously distributed flux lines in contact with the bottom surface of the insert, in the dynamic condition.

The chip was fabricated by rapid prototyping procedure, and it was characterized by two PDMS, (Sylgard 184, Mascherpa) blocks, the upper and the lower, bonded to each other and in turn attached to a glass slide as support. Figure 1A,B shows the design and the picture of the upper and lower block of the chip separated and bonded together. The upper block (50 × 30 × 3 mm) presented a central space, obtained by using a hollow cutter, in which a porous insert was placed. For all the experiments, we used porous inserts having 0.4 μm porosity, as typical for human bronchial epithelia cultured at the ALI.^22−25^ The central space of the upper block can be made with a different diameter (e.g., 6.5 or 12 mm) to accommodate different kinds of porous inserts (e.g., Transwell 3470 or Snapwell 3801, as shown in Supporting Information S1). Video S2 shows the simple insertion of the Transwell into the microfluidic chip. The lower block (50 × 30 × 2 mm) presented a microfluidic channel (15 × 4 × 1.5 mm) and a hexagonal pool in the center (surface of 2.08 cm^2^ and volume of 0.312 cm^3^). Both the blocks of the device were prepared by demolding PDMS, from a poly(methyl methacrylate) (PMMA, Goodfellow) stamp. PMMA masters were designed by AutoCAD software generating a CAD file, which was subsequently converted into a CAM format by using Deskam. The CAM file was read by a micro-milling machine (Minitech CNC Mini-Mill), and a positive relief geometry was printed by digging on the PMMA slab. The PDMS prepolymer was mixed with the curing agent in a 10:1 (w/w) ratio, centrifuged for 5 min at 3000 rpm, poured on the PMMA master, and vacuum degassed. The PDMS-PMMA system was incubated at 80 °C for 60 min to allow PDMS polymerization. Afterward, the PDMS was peeled off from the PMMA master. A Au wire, 0.33 mm (Goodfellow), was inserted into a groove dug into the PDMS, and the top and bottom blocks were irreversibly assembled using a small amount of PDMS and bonded at 80° for 60 min. Finally, a very thin PDMS layer was used to seal inlet and outlet pipes and attach the chip on a glass slide. The chip was incubated at 60 °C overnight to achieve irreversible bonding. Here, we used PDMS because it remains the most popular material for lab-on-a-chip technology and other biomedical applications due to its low cost, ease of fabrication, elasticity, oxygen permeability, biocompatibility, and optical transparency. To reduce PDMS hydrophobicity and reduce undesired interactions between small molecules and PDMS,^26−32^ we performed an O_2_ plasma treatment for 1 min (50 W), and then, we added water into the channel of the device to avoid the reversion of the reaction after exposure to the air. The protocol we used to reduce PDMS hydrophobicity was previously validated by the literature. The oxygen plasma treatment induces changes in surface wettability, thanks to the exposure of the silicone surface to hydrophilic −OH groups. B. Jiang et al. demonstrated a decrease in PDMS surface hydrophobicity after oxygen plasma treatment due to hydroxyl groups speeding up the process of water droplet dispersal on the PDMS surface. Contact angle analysis highlighted that the PDMS surface became highly hydrophilic when the oxygen plasma treatment time was over 1 min.^33^ Furthermore, the plasma treatment we used here (1 min, 50 watts) was the same adopted in previously published papers for PDMS bonding,^14,34^ which relies on the contact between hydrophobic PDMS layers.^35^ Afterward, the chip was sterilized with UV light before performing the dynamic cell culture. To this aim, tubes and connectors were used to establish a closed circuit between a medium reservoir and the chip through a peristaltic pump operating at a 40 μL/min flow rate (Video S3 and Supporting Information S4 show the entire setup of dynamic culture for single or multiple chips). The dynamic setting comprising the chip and the peristaltic pump was placed into a conventional CO_2_/temperature-controlled incubator (5% CO_2_ and 37 °C) to let the epithelium grow. 8 mL of culture medium was added into the reservoir, and the medium was changed twice a week.

### Mathematical Model CFD Simulation

To define the dynamic experimental setup, we used the commercial CFD COMSOL Multiphysics version 5.0. Concerning the boundary conditions, at the chip, outlet atmospheric pressure was considered (*p* = 1 atm), and a no-slip condition was adopted at the walls. To simulate culture conditions, the laminar flow and diluted species transport modules in steady-state conditions were coupled. Different flow rates were applied at the inlet to evaluate the best culture conditions in terms of oxygen supply and shear stresses. For the single-phase fluid flow, the laminar flow physics implements the Navier–Stokes equations for the conservation of momentum (1) and the continuity equation for the conservation of mass (2). The modeled liquid was incompressible, and the governing equation in the fluid region was given by the following equations
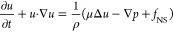
1
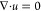
2where *u* = (*u*,*v*)^*T*^ is the velocity vector, *p* is the hydrodynamic pressure, ρ is the density, *f*_NS_ is the external body force, and μ is the dynamic viscosity coefficient. The diluted species transport physics implements the mass balance equation of the oxygen diluted in the culture medium (3). The oxygen concentration within the system was calculated using the following mass balance eq 3
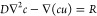
3where *c* is the oxygen concentration, *D* is the diffusion coefficient of the oxygen, and *R* is the volumetric oxygen consumption rate expressed by the Michaelis–Menten law and according to the next eq 4
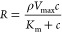
4where *V*_max_ is the maximum oxygen consumption rate, *K*_m_ is the concentration at which the oxygen consumption rate is half of *V*_max_, and ρ is the cell density in the cultivation chamber obtained by taking into account the number of cells present in the HBE housed into the Transwell fitted in the microdevice. *R* was set to 0 in the fluid domain, assuming that only the cells consumed the oxygen. Based on experimental and literature data, the CFD simulation parameters are indicated in Table 1.

**Table 1:** CFD Simulation Parameters

property	variable	expression	unit	references
O_2_ concentration	*C*_O_2__	0.22	mol/m^3^	(16)
O_2_ diffusion coefficient	*D*	3 × 10–^9^	m^2^/s	(36)
cell density	ρ	7.5·10^13^	cell/m^3^	(37)
maximum velocity of O_2_ consumption	*V*_max_	3.42 × 10–^16^	mol/(cells·s)	(38)
O_2_ concentration at *V*_max_/2	*K*_m_	2.14 × 10–^1^	mol/m^3^	(38)

The initial condition for O_2_ concentration in the culture medium is 0.22 mol/m^3^, the diffusion coefficient (*D*) is 3 × 10^–9^ m^2^/s, cell density (ρ) is 7.5·10^13^ cell/m^3^, maximum velocity of O_2_ consumption (*V*_max_) is 3.42 × 10^–16^ mol/(cells·s), and O_2_ concentration at *V*_max_/2 (*K*_m_) is 2.14 × 10^–1^ mol/m^3^, and the convection–diffusion equation was implemented in the model to evaluate the O_2_ consumption in the bio-system. Finally, another important parameter that contributed to the choice of the proper flow rate was the shear stress. In the simulation, the study of the shear stress was integrated, based on the velocity results coming from the steady-state study. The modeled fluid corresponded to the culture medium that can be easily approximated to water, so a Newtonian fluid, according to eq 5
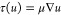
5where μ is the dynamic viscosity of the flow and *u* is the flow velocity along the boundary.

### Tissue On-Chip Culture

Transwells were coated with 10 μg/mL rat tail collagen for 30 min in the incubator. Human bronchial epithelial cells were seeded into the coated Transwell inserts (250,000 cells for the insert of 6.5 mm diameter) in LHC/RPMI medium, as reported previously.^22,23^ On the day after the seeding, the medium was replaced with PneumaCult-ALI maintenance medium (stem cell), and tissue was cultured at the ALI for 14 days in static or dynamic conditions. For the static culture, the Transwell was placed into a 24-multiwell; meanwhile, for the dynamic culture, the Transwell was moved into the chip. The dynamic condition of the microfluidic chip was settled with a peristaltic pump (*Q* = 40 μL/min). The medium flowed in the basal side of the porous insert; meanwhile, the apical part was exposed to the air, to obtain the ALI condition on-chip. The culture medium in the reservoir was changed twice a week. After 14 days, human bronchial epithelia were analyzed to evaluate the presence of cilia and mucus by immunofluorescence, vitality (by lactate dehydrogenase (LDH) assay, Sigma-Aldrich, according to the manufacturer protocol), ciliary beat frequency, and mucus viscosity [by multiple particle tracking (MPT)]. Response to treatment with 3 μM Lumacaftor (VX-809 Selleckchem) at the basolateral side of the sample (*n* = 3) was evaluated by analyzing mucus viscosity and thickness (by the aerosol of the fluorescent nanoparticle). Electrical measurements (by a potentiostat) were performed at 3, 7, and 14 days of culture.

### Scanning Electron Microscopy

Samples were fixed with 2.5% glutaraldehyde or perfluorocarbon (1% OsO_4_ in perfluoro-compound FC-72 Acros Organics) followed by critical point drying (CPD-300 Leica). Then, they were mounted onto metal stubs and then coated with a 100 A ultrathin gold layer (thickness 7–15 nm) in a glow-discharge coater to minimize charging and increase the conductivity of the biological material (sputter coater Cressington_HR 208). Images were acquired using an InLens detector [scanning electron microscopy (SEM), FEG_Ultrapluss by ZEISS].

### Fluorescence Analysis

Samples were directly fixed in 4% paraformaldehyde (Sigma-Aldrich) overnight at 4 °C. The sample washed before fixation was avoided to circumvent excessive mucus dilution and keep the differences in mucus properties in all the conditions. After the fixation samples were washed once with PBS (1X) and permeabilized using Triton (0.1%), they were diluted with PBS for 5 min at room temperature (RT). At this point, the blocking solution, composed of 3% BSA, 3% FBS, and 0.01% Triton w/v all diluted in PBS, was added to the samples for 1 h at RT. Samples were incubated overnight with primary antibodies diluted with the blocking solution: anti-MUC5AC (Abcam, ab78660, produced in rabbits and used at 1:100 dilution) and anti-acetylated tubulin (Abcam, ab15246, produced in rabbits and used at 1:100). After incubation with primary antibodies, samples were washed with PBS and then incubated with a mixture of secondary fluorophore-conjugated antibodies (AlexaFluor) for 1 h at RT, in the dark. Later, samples were washed and stained with a fluorescent phalloidin (Invitrogen 1:200) to mark the actin (40 min at RT) and with DAPI (Sigma-Aldrich) to mark the nuclei (20 min at RT). Samples were observed using a confocal microscope (Confocal Leica TCS SP5 II). Images were acquired with a resolution of 12 bit, 1024 × 1024 pixel by using a 25× water immersion objective (HCX IRAPO L 25.0 × 0.95 Water, n. a. 0.95). Image analysis was performed with ImageJ. To estimate the quantity of mucus and cilia per cell, the area relative to the fluorescent protein signal (MUC5AC and β-tubulin) was thresholded, measured (in μm2), and divided for the number of cells obtained by counting cell nuclei stained by DAPI with “analyze particles” (*n* = 10). The wand tracing tool was used to select single cells from thresholded images, and the mean morphological parameters (area, aspect ratio, and circularity) were calculated for each condition (*n* = 20).

### TEER and Capacitance Measurements

1 h before the analysis, the culture medium in the reservoir was replaced with basal medium and left to stabilize in contact with the cells. Electrical measurements were performed by placing the dynamic setting under the biological hood and connecting the Au electrode in the basal side of the microfluidic device to an AUTOLAB PGSTAT302N (potentiostat/galvanostat) equipped with a FRA32M module (frequency response analysis module). An additional electrode was placed in the apical part of the Transwell, perpendicular to the basal electrode. This external electrode was fixed on a base to keep the height constant and avoid damaging the epithelium. Both the basal and apical electrodes were embedded in the basal medium equilibrated at 37 °C for measurements. The impedance spectra were recorded in the potentiostatic mode (0.3 V) with an amplitude of 0.01 V and frequencies ranging from 100 kHz to 0.1 Hz between two Au electrodes. For each measurement, three readings and 50 data points (logarithmic frequency step) per reading were collected. Bode the modulus and impedance at 12 Hz were used to monitor the epithelium growth as reported in the literature. TEER and capacitance parameters were obtained by fitting experimental impedance data with a typical electric circuit representing epithelia. Specifically, this circuit was composed of a resistance and a capacitance in parallel, representing the tight junctions and the lipid bilayer of the plasma membrane. Furthermore, the circuit takes into account the resistance of the surrounding medium and the capacitance of the electrodes that were added in series into the circuit.^39^

### Ciliary Beat Frequency

Ciliary beat frequency on differentiated epithelia was acquired by using an inverted fluorescence motorized microscope (Zeiss Axio Observer 1) at a frequency of 100 frames/second, 20 air objective with 1.6 aperture. Videos were analyzed by using ImageJ. They were converted to grayscale, and a region of interest (ROI) on cilia was selected and cropped. Cilia beating was analyzed by using Multi kymograph plug-in and plot profile of ImageJ. The number of visualized picks represented the ciliary beat that was subsequentially normalized considering the frequency of acquisition to obtain the ciliary beat frequency.

### Microrheology by MPT

48 h before mucus collection, the apical epithelial surface was washed with PBS. 120 μL of the same solution was left in the apical part of the Transwell and later harvested, as previously described.^40^ For MPT experiments, carboxyl-modified fluorescent nanoparticles (200 nm diameter, Polysciences, Inc.) in a 2% (w/v) suspension were added to the mucus withdrawn from the surface of the sample at a final concentration equal to 0.01%. The sample was sandwiched on a 35 mm FluoroDish and covered with a glass slide to avoid evaporation. Before starting the acquisition, the sample was kept at RT for 15 min to allow the mucus to equilibrate and relax. The setup was placed on an active optical table to reduce the effect of vibrations. The motion of nanoparticles embedded in at least 8 different regions of the mucus was recorded for a total of 5 s at 20 frames per second using an inverted fluorescence microscope (Olympus IX81; Olympus) equipped with a 60× water immersion objective (N.A. = 1.20) and a Hamamatsu ORCA-Flash 2.8 CMOS camera (Hamamatsu) in at least 8 different regions. By using our self-developed Matlab code, the point-tracking trajectories are generated in two distinct steps: first, the beads are detected in each frame, and then, the points are linked into trajectories. Each position is determined by intensity measurements through its centroid, and it is compared frame by frame to identify the trajectory for each particle, based on the principle that the closest positions in successive frames belong to the same particle (proximity principle). Once the particle trajectories are obtained, mean squared displacements (MSDs) are calculated by using the following equation
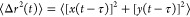
6where angular brackets mean time average, τ is the time scale, and *t* is the lag time.

The average MSD of all nanoparticles belonging to the same region was used to derive the mucus viscosity by applying one of the two different approaches described below.

In general, for viscoelastic fluids, a power law dependence of the MSDs on the lag time *t* is observed
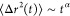
7

When the scaling exponent α approximates 1 (α > 0.95), the mucus behaves as a purely viscous fluid and the eq 7 becomes
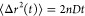
8where *n* is the number of dimension (here equal to 2) and *D* is the diffusion coefficient which is related to the mucus viscosity by the Stokes–Einstein equation^41^
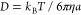
9where η is the viscosity, *a* is the sphere’s radius, *k*_B_ is the Boltzmann’s constant, and *T* is the absolute temperature (*k*_B_*T* is the thermal energy).

When the scaling exponent is lower than 1 (α > 1), the mucus behaves as a viscoelastic fluid. By using a modified algebraic form of the generalized Stokes–Einstein equation,^42^ the MSDs can be converted to the time-dependent creep compliance *J*(*t*)
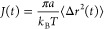
10Then, by using the Maxwell model for the viscoelastic mucus behavior, the creep compliance *J*(*t*) is further related to both mucus viscosity η and elastic modulus *G*(43)
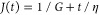
11

### Detection of Mucus Thickness by Aerosol

48 h before the experiment, samples were washed 3 times with PBS1x to remove the previously secreted mucus. Afterward, samples were exposed at the ALI for 48 h. In this way, it was possible to evaluate the thickness of the mucus released by the cells and accumulated on the surface. To detect the thickness of the apical mucus, the nuclei of the epithelium were marked by vital nuclear stain with Hoechst (Invitrogen, 1:10.000) added in the basolateral side of the sample for 1 h at 37 °C into the incubator. Later, (vol/vol) 0.1% fluorescent nanoparticles (500 nm diameter, Polysciences, Inc.) were aerosolized on the apical side of the epithelia (Aerogen Solo nebulizer). Samples were not washed before particle nebulization to avoid mucus dilution. Aerosolization of the fluorescent nanoparticles on the surface of the mucus was performed at RT and required 30 s. Immediately after the nebulization, *Z* stack acquisitions of live samples were performed by using the Confocal Leica TCS SP5 II (25× water objective), and 3D views were obtained with ImageJ.

### VX809 Administration via Aerosols

We delivered the drug at a concentration of 60 μM, a value higher than that used when VX809 was delivered by the basolateral route, in order to assure the same number of molecules in both conditions:

- Static: 3 μM VX809; basolateral volume: 2 mL; 0.006 μmol.
- Aerosol: 60 μM VX809; apical delivered volume: 100 μL; 0.006 μmol.

We experimentally evaluated that the aerosol delivered about 3–4 μL per second, and, consequently, we aerosolized 100 μL of VX809 solution at 60 μM (diluted in the culture medium) for 30 s on the apical side of the sample. As a negative control, we aerosolized a solution with DMSO diluted in the culture medium on the surface of the CF sample because DMSO is the diluent of the stock solution of VX809 (Sellenchem cat. no. S1565, containing 1 mL of 10 mM VX809 in DMSO).

### Statistical Analysis

Cells from two CF (BE115 and BE86) and two non-CF (BE122 and BE37) were used for the experiments that were performed in triplicate. For image analysis, at least 10 images were analyzed for each sample. Graphics show data expressed as mean ± SD. Differences between groups were determined using the statistic test ANOVA Tukey HSD test for all the comparisons except for mucus viscosity where the distributions were not normal and were analyzed by the Kruskal Wallis test. Significance between groups was established with a *p value <0.05, **p value < 0.01, and **p value < 0.001.

## Results and Discussion

### Optimization of Fluid Dynamic Conditions for Differentiating Human Bronchial Epithelia On-Chip

**Figure 2:**
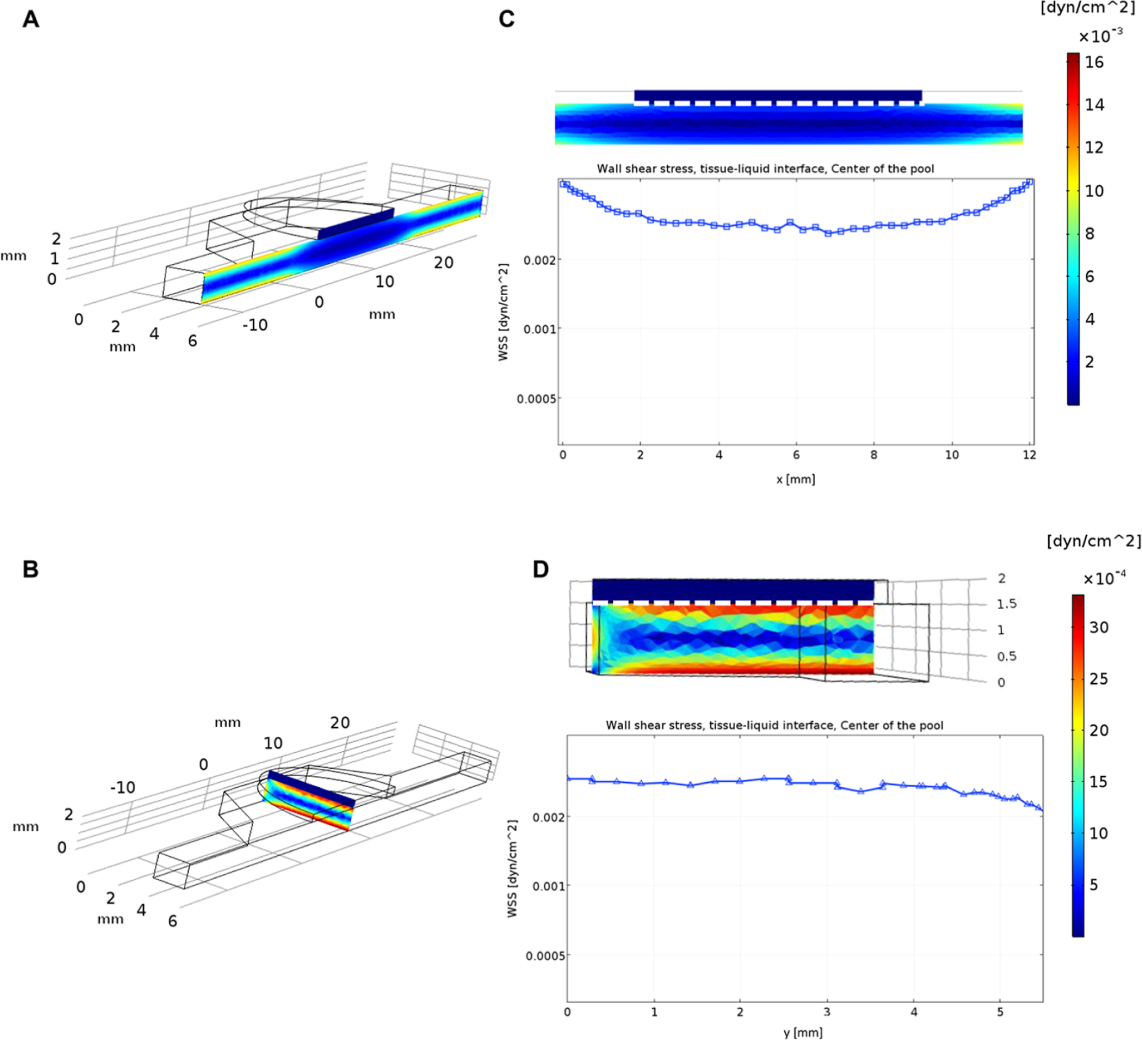
COMSOL simulation of shear stress: (A) colormap of shear stress along the *x* direction of the chip; (B) colormap of shear stress along the *x* direction of the chip; (C) zoom-in on the tissue–liquid interface in the *x* direction and graphic of shear stress values; the colormap legend on the right reports the range of shear stress in the *x* direction; and (D) zoom-in on the tissue–liquid interface in the *y* direction and graphic of shear stress values; the colormap legend on the right reports the range of shear stress in the *y* direction.

The microfluidic device was fabricated to host commercial porous inserts (such as Transwell or Snapwell) and allow, in a very simple manner, the dynamic culture and the live monitoring of CF bronchial epithelia. The chip presented a top and a bottom block bonded to each other. Figure 1A,B shows the design and the macroscopic pictures of each block and the bonded chip. The device was also attached to a glass slide to enhance the stability of the system (Figure 1A). The top block contained a central space for insert accommodation. Video S2 demonstrates the simplicity of the insertion of a Transwell into the chip. Instead, the bottom part included a channel for medium flow from the entrance (inlet) to the exit (outlet) of the chip and a central hexagonal pool (Figure 1A,B). A gold electrode was added between the top and the bottom part during the bonding (Figure 1A,B) to enable the online monitoring of tissue electrical properties by impedance analysis. The microfluidic chip was connected to a reservoir and a peristaltic pump by using tubing and connectors. This allowed the establishment of a closed-loop circuit for the dynamic culture of the cells seeded on the porous insert (Video S3 and Supporting Information S4). Multiple chips could be connected to the same peristaltic pump in parallel (Supporting Information S4). Fluid dynamic simulations were performed to establish the right geometry of the chip and the flow rate able to sustain tissue growth and differentiation. For the dynamic culture, we used the geometry indicated in Figure 1A,B and a flow rate of 40 μL/min. Chip geometry and flow dynamic conditions were adjusted to guarantee tissue vitality and differentiation. The final choice of the hexagonal geometry of the pool was the result of a series of experimental trials to identify the best geometry of the chip. The presence of the central hexagonal pool in the lower block of the chip was necessary to slow down the flow and make nutrient supply homogeneously available at the basal side of the insert. At the same time, channel dimensions (15 × 4 × 1.5 mm) were optimized to guarantee homogeneous shear stresses and flux lines. Figure 1C shows the results of the COMSOL simulation by setting the flow rate at 40 μL/min in terms of tissue oxygenation, velocity, and shear stress. In this condition, the oxygen concentration in the chip was 0.2 mol/m^3^, as indicated by a previous work underlying the importance of microfluidics for faster epithelial differentiation.^14^ Flow velocity under the porous membrane was 1 × 10^–5^ m/s, similarly as indicated by other models of airways-on-chip.^17,18^ The system provided low shear stresses in the basal side of the epithelium with a homogeneous distribution of the flux lines. Figure 2 indicates that the distribution of shear stresses in the pool of the chip was constant and homogeneous at the tissue–liquid interface, both along the *x* and *y* directions. Representative shear stress sections are reported in Figure 2A,B (respectively, along the *x* and *y* direction). Zoom-in on sections and shear stress distributions are shown in Figure 2C,D. The values of shear stress in the graphic were calculated along the dashed white line, representing the tissue–liquid interface, in the *x* and *y* directions (Figure 2C,D). Whatever the insert is, shear stresses are the same from the perimeter to the center. All values along *x* and *y* were around 0.0025 dyne/cm^2^ (Figure 2C,D), showing a uniform distribution at the tissue–liquid interface on the entire surface for cell seeding. The value of shear stress on-chip was lower than that in vivo, where it was about 0.5–1 dyne/cm^2^.^44^ However, vessels in vivo are embedded into an extracellular matrix (ECM) which, except at the level of the alveoli, is interposed between the vessels and the epithelium, cushioning the shear stress from blood vessels. In fact, the ECM applies a high flow resistance, which significantly slows down the interstitial flow and reduces shear stress.^45,46^ At the same time, other works demonstrated that the use of low but homogeneous shear stresses facilitates tissue differentiation and reduces cell damage.^47,48^

As expected, fluid dynamic parameters widely change by modifying the geometry of the chip. For example, Supporting Information S5 shows how the decrease of the fluidic channel dimensions (from 15 × 4 × 1.5 mm to 15 × 1 × 1.5 mm) impacts shear stress distribution and tissue differentiation, by setting the same flow rate of 40 μL/min. Indeed, in such conditions, heterogeneously distributed flux lines were generated on the bottom of the tissue (Supporting Information S5A), thus inducing a heterogeneous differentiation of the cells of the epithelium with a lack of epithelial integrity and non-measurable TEER. Figure S5 shows the distribution of the shear stress along the *x* (12 mm) and *y* (6 mm) directions of the central pool of the chip (Supporting Information S5B), considering these two different channel dimensions (15 × 4 × 1.5 mm and 15 × 1 × 1.5 mm). In the chip with larger channels, the values of shear stress along the *x* and *y* directions were very close to each other (about 0.0025 dyn/cm^2^), indicating the shear stress homogeneity at the tissue–liquid interface (Supporting Information S5C). Instead, in the chip with smaller channels, the values of shear stresses along *x* and *y* were different, resulting in a non-homogeneous distribution at the tissue–liquid interface (Supporting Information S5D). As a consequence, epithelial cells cultured in the device with smaller channels presented heterogeneous differentiation, a lack of epithelial integrity, and non-measurable TEER. The image reported in Figure S5F is an example of a field of a non-well differentiated epithelium obtained by using the device with smaller channels. In this case, the value of TEER resulted negative and, consequently, non-realistic. Indeed, for the measurement of the TEER, we fitted results from impedance analysis with a circuit which requires the presence of an intact and continuum epithelium. Non-measurable values of TEER and fluorescence analyses were both evidence of the lack of a barrier epithelium. For this reason, we adjusted the chip geometry to obtain homogeneous shear stresses and we underline the critical role of this fluidic parameter to obtain fully differentiated epithelia with barrier function, as demonstrated in the next paragraphs. To evaluate cell viability on-chip, we quantified the activity of the LDH released in the culture medium. It is an indirect index of cell vitality because injured cells release the enzyme proportionally to their damage.^15^ LDH activity measured at day 14 (4 days after the change of the culture medium) was very low, both in static and dynamic conditions, indicating the absence of toxicants in the culture (Supporting Information S6). However, the activity of the LDH in the static condition was higher, thanks to dynamic fluidic conditions.

### CF Human Bronchial Epithelia Differentiated On-Chip with a Higher Presence of Cilia and Mucus Compared to the Static

**Figure 3:**
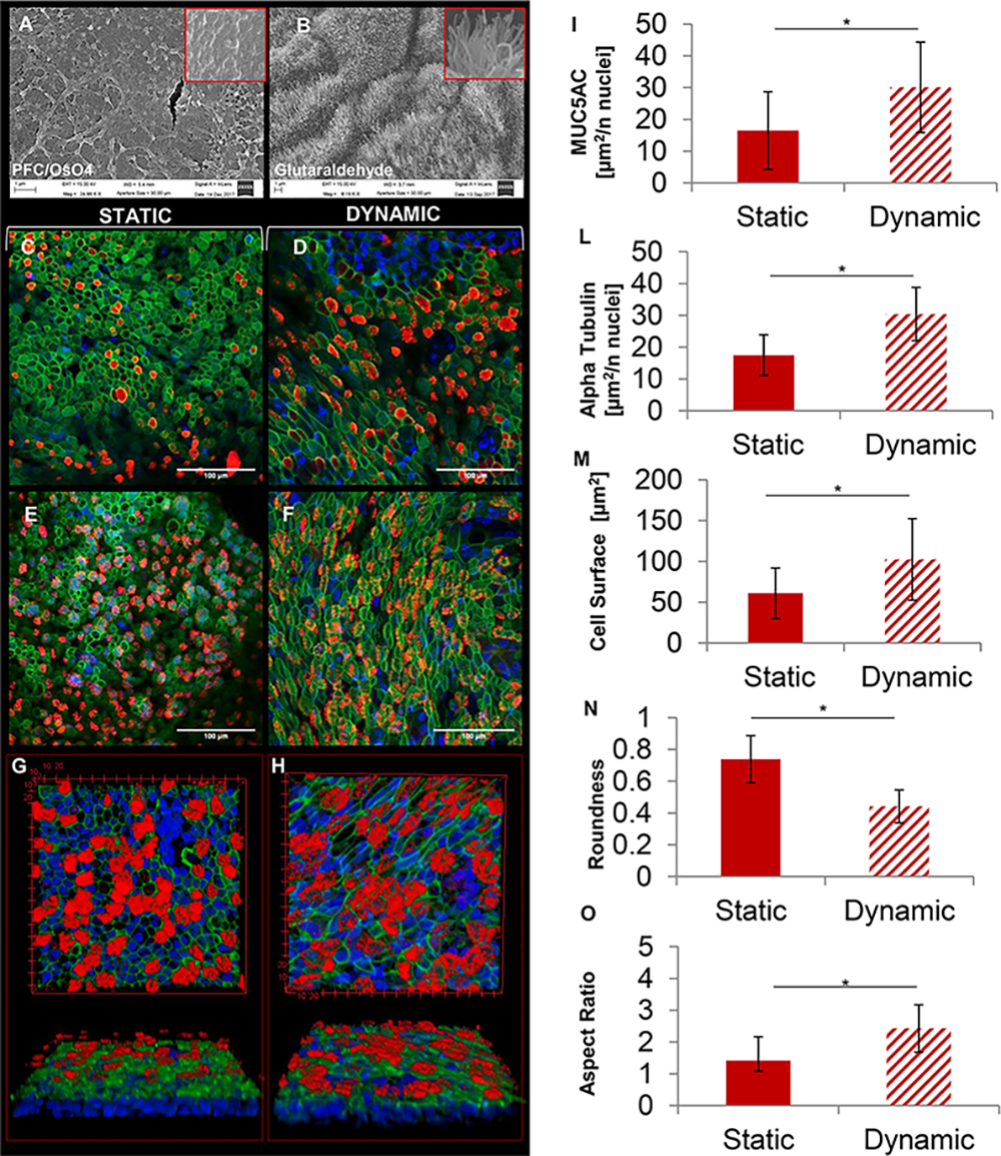
Morphology of human CF bronchial epithelium on-chip: (A) SEM image of the mucus present in the apical part of the sample fixed with PFC; (B) SEM image of the cilia present in the apical part of the sample fixed with glutaraldehyde; (C/D) immunofluorescence images showing the mucine MUC5AC in red, the actin marked by phalloidin in green, and nuclei marked by DAPI in blue, in static (C) and dynamic (D); (E/F) immunofluorescence images showing α-tubulin in the cilia in red, actin marked by phalloidin in green, and nuclei marked by DAPI in blue, in the static (E) and dynamic (F); (G/H) 3D view showing α-tubulin in the cilia in red, actin marked by phalloidin in green, and nuclei marked by DAPI in blue, in the static (G) and dynamic (H); (I) quantification of the MUC5AC signal (μm^2^/number of nuclei) in static vs dynamic; (L) quantification of the α-tubulin signal (μm^2^/number of nuclei) in static vs dynamic; (M) quantification of cell surface (μm^2^) in static vs dynamic; (N) quantification of cell roundness in static vs dynamic; and (O) quantification cell aspect ratio in static vs dynamic; (I/O) bar plots report mean values ±SD. *p value <0.05; (A/B) scale bar 1 μm; (C/D/E/F) scale bar 100 μm. The figure is related to CF cells (images from the BE115 primary cells), for image analysis, *n* = 10.

CF human bronchial epithelia (HBE-CF) differentiation after 14 days of ALI culture on-chip was demonstrated by the presence of apical mucus and cilia. SEM images in Figure 3A,B were obtained by observing the apical side of the epithelium after fixation by perfluorocarbon (PFC/OsO4) or glutaraldehyde, respectively. PFC is an anhydrous fixative able to completely preserve airway surface liquid and mucus, which instead are mainly diluted and removed by using aqueous solutions. For this reason, Figure 3A shows the mucus preserved on the surface of the epithelium after fixation by PFC/OsO4. In contrast, Figure 3B highlights the presence of the cilia on the surface of the sample after the removal of the mucus by washing with PBS and fixation with glutaraldehyde. Fluorescence images and graphics from image analysis in Figure 3 demonstrate that the dynamic fluidic stimulus enhanced the presence of cilia and mucus on the HBE-CF. Figure 3C,E,G shows the presence of the mucus (marked by MUC5AC) and cilia (marked by the α-tubulin) in the static condition. Instead, Figure 3D,F,H is related to the dynamic condition on-chip. The plots obtained by image analysis in Figure 3I,L underline statistical differences between HBE-CF cultured in static and dynamic conditions. We performed image analysis on α-tubulin immunofluorescence images to quantify the surface extension, resulting positive for the α-tubulin signal, and we normalized this value to the number of cells in the ROI obtained by counting the cell nuclei marked by DAPI. Therefore, we hypothesized that an increase in this ratio means an increase in the number of cilia per cell. Moreover, we did not observe significant changes in the number of cell nuclei per ROI in the static vs dynamic condition. We observed morphological differences between the static and dynamic cultures. CF cells cultured on-chip showed a more elongated shape and a higher cell surface (Figure 3H,I), resulting in a higher aspect ratio and decreased roundness compared to cells cultured in static conditions (Figure 3L,M). This phenotypic change demonstrated that the cells felt the effect of the basal shear stresses in dynamic conditions and reorganized their cytoskeleton according to the physical stimulus.

### CF Epithelia Showed Different Features in Comparison to Non-CF Bronchial Samples On-Chip

**Figure 4:**
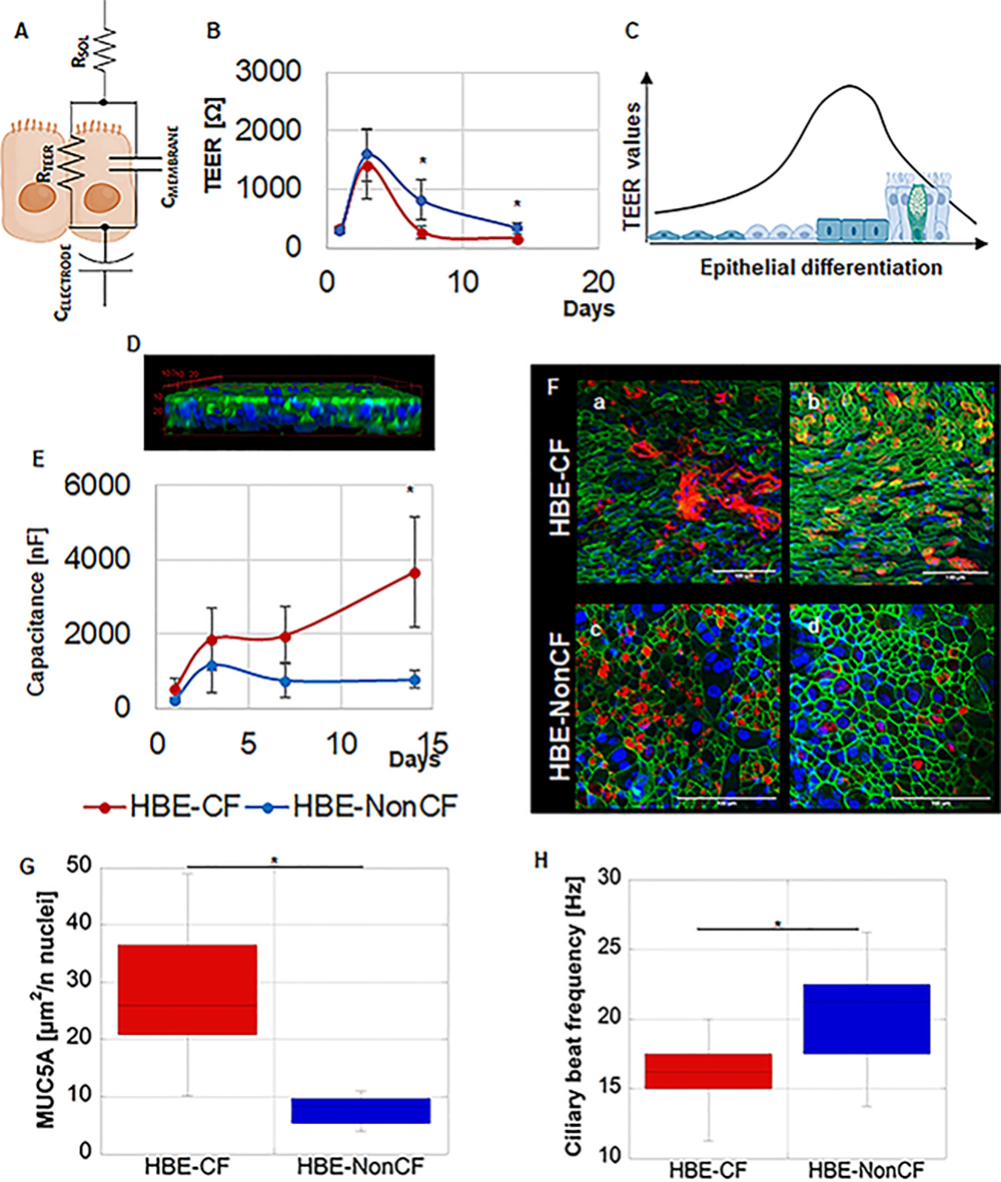
Differences between CF epithelium vs non-CF epithelium on-chip: (A) graphical representation of the electrical circuit of the epithelium; (B) graphic of TEER measurements (Ω) over time culture (days), the graph reports mean values ±SD; (C) design representing the changes occurring in TEER values during epithelial differentiation in vitro, created with BioRender.com; (D) 3D view of the differentiated epithelium with nuclei marked in blue by DAPI and actin marked in green by 488-phalloidin; (E) graphic of capacitance measurements (nF) over time culture (days), the graph reports mean values ±SD; (F) immunofluorescence showing MUC5AC (a,c) and α-tubulin (b,d) in red; cell actin is marked in green by Phalloidin and cell nuclei in blue by DAPI; (a,b) HBE-CF samples, (c,d) HBE-NonCF samples; scale bar 100 μm; (G) box plot from the image analysis of the MUCAC (μm^2^/number of nuclei) in CF vs non-CF epithelium on-chip; and (H) box plot of the ciliary beat frequency (Hz) of CF vs non-CF epithelium on-chip; *p value <0.05. The figure represents the results from CF cells (both BE86 and BE115 primary CF cells) in comparison with nonCF cells (both BE122 and BE37 primary nonCF cells). Electrical measurements were performed on BE86 and BE115 primary CF cells and BE122 and BE37 primary nonCF, *n* = 10. Image analysis was performed on BE115 CF cells and BE122 nonCF (for mucus quantity and ciliary beat frequency).

To still investigate tissue differentiation over time and highlight differences between CF and non-CF samples, we cultured both CF and non-CF human primary bronchial epithelial cells on different chips in parallel. We performed live analyses such as the measurement of tissue electrical properties and evaluated the ciliary beat frequency. Moreover, we fixed the samples and analyzed the mucus on the surface of the sample by immunofluorescence for MUC5AC. Thanks to the presence of the gold electrode in the microfluidic chip, we were able to perform the online monitoring of tissue electrophysiological properties over culture time. The gold electrode was placed in the middle of the hexagonal pool and on the bottom side of the porous insert housing the bronchial epithelial model. Measurements were carried out by adding a second electrode on the top of the insert, perpendicular to the first one (Supporting Information S7A), and connecting both electrodes to a potentiostat (Supporting Information S7B). Impedance spectra were recorded for each time point (3, 7, and 14 days). These spectra were analyzed by fitting with an equivalent circuit, as shown in Figure 4A. The circuit was based on biological, physical, and chemical evidence, indicating the tight junction between adjacent cells as an Ohmic resistance (*R*_teer_) and the cell membrane as a capacitor (*C*_membrane_). Moreover, the circuit comprised the resistance of the culture medium (*R*_sol_) and the capacitance of the electrodes (CE), similarly as previously described by Benson et al.^39,49^ We reported the measure of *R*_teer_ in Ω and capacitance in nF, as previously reported in a microfluidic chip with integrated electrodes, and compared the measures obtained into the same chip over time culture.^50^ Impedance analysis was directly performed on-chip without the need to move the dynamic sample. Instead, static samples were moved into a microfluidic chip for measurements. After 3 days of dynamic culture, both HBE-CF and HBE-NonCF samples reached a peak value of the *R*_teer_ (Figure 4B). This peak was significantly higher than the corresponding samples in static, thus demonstrating a faster differentiation on-chip. Table S8 shows *R*_teer_ measurements in static and dynamic for HBE-CF and HBE-NonCF at day 3 of culture. As reported in the literature and schematically represented by the design in Figure 4C, the peak of *R*_teer_ values should indicate the moment of the maximum strength of epithelial tight junctions, whereas the subsequent decrease indicates cell polarization.^51,52^ The decrease of the *R*_teer_ suggested a loosening of tight junction strength after day 3 and the start of cell polarization. The same trend of *R*_teer_ over time was observed for HBE-CF and HBE-NonCF (Figure 4B). Furthermore, *R*_teer_ values were lower in HBE-CF vs HBE-NonCF (with statistically relevant differences on days 7 and 14). This was in accordance with previous results, showing reduced barrier function in CF epithelial cell monoculture^53,54^Supporting Information S9 shows the differences between the trend of *R*_teer_ in dynamic and static. The peak of *R*_teer_ was not detectable in static (Supporting Information S9A). At the same time, the presence of cilia and mucus increased and was enhanced in dynamic vs static, after day 3 (Supporting Information S9A,B). In fact, the presence of cilia and mucus was already detected at day 3, but no morphological differences in mucus/cilia quantity were observed, although electrical measures highlighted differences between dynamic and static. At day 7, HBE-CF samples showed enhanced mucus and cilia quantity in dynamic vs static. Differences were more evident at day 14. The 3D view of the HBE-CF in Figure 4D (14 days) highlights the presence of cell nuclei in different positions of the *z* plane, as typical of pseudostratified epithelia. From impedance spectra, it was also possible to achieve capacitance measures. The biological principle behind this analysis is that the plasma membrane acts as a capacitor whose capacitance depends on the distance between the conductors and their lengths such as the thickness of the bilayer and the extension of the cell surface.^55^ At the microscopic level, this parameter depends on the different components of the cell membrane in terms of proteins, lipids, and carbohydrates, as well as exocytic/endocytic processes. At the macroscopic level, different cell shapes are associated with different degrees of cell membrane extension and deformation, reflecting changes in cell/tissue capacitance. Capacitance values were higher in the HBE-CF than in HBE-NonCF, especially after 14 days of culture (Figure 4E). These changes matched with fluorescent images, indicating an elongated cell shape in HBE-CF but not in HBE-NonCF (Figure 4F). The different morphologies of HBE-CF but not in HBE-NonCF were in accordance with already reported changes in the cell shape in CFTR-defective epithelia.^53^ Moreover, higher capacitance values in CF may be related to the increase in mucus secretion in HBE-CF. In this regard, Danahay et al. already showed the parallel increase of tissue capacitance and mucus secretion from airway epithelia.^56^ The quantity of mucus on the surface of the HBE-CF was higher than that of HBE-NonCF (Figure 4F(a,c) and Figure 4G), and the ciliary beat frequency was lower in HBE-CF vs HBE-NonCF (Figure 4H). This latter result was expected due to the known impairment of the airway surface liquid and mucus clearance in the pathological state.^57^ Thanks to the possibility to monitor the electrophysiological properties of the live tissue, another possible application of capacitance measurements on-chip may be the study of CFTR translocation into the apical cell membrane in response to chemical stimuli. Previous works demonstrated that the functional insertion of CFTR from cytoplasmic vesicles into the plasma membrane can induce an increase in cell capacitance due to the extension of the cell membrane.^58,59^Supporting Information S10A illustrates the principle of surface increase after the integration of vesicles containing CFTR in the apical side of the cell membrane. In this context, we produced preliminary data stimulating both HBE-CF and HBE-NonCF with the cAMP activator forskolin (20 μM forskolin added both in the apical and basolateral side of the sample). Already after 3 min, we registered an increase of the capacitance in the normal (HBE-NonCF) but not in the pathological samples (HBE-CF) (Supporting Information S10B,C). Moreover, after stimulation with forskolin, we observed CFTR apical localization in HBE-NonCF but not in HBE-CF (Supporting Information S10D,E). Despite this, we observed an experimental variability in cell capacitance change after forskolin induction, maybe related to different disposability of CFTR-containing vesicles behind the plasma membrane and the activation of other exocytic/endocytic processes regulating cell surface extension.^60,61^ The optimization of the mentioned procedure may be useful to investigate the efficacy of drugs aiming at increasing the functional CFTR trafficking to the plasma membrane. We expect that the real-time monitoring of capacitance change may give insights into the functional trafficking of CFTR to the cell membrane, similarly as reported by the transient capacitance increase evoked by cAMP stimulation in *Xenopus* oocytes expressing CFTR due to the increase of the membrane surface after integration of CFTR from sub-membrane vehicles into the bilayer.^58^

### Proof of Principle of Drug Administration On-Chip: VX-809 Reduced the Mucus Viscosity and Thickness

**Figure 5:**
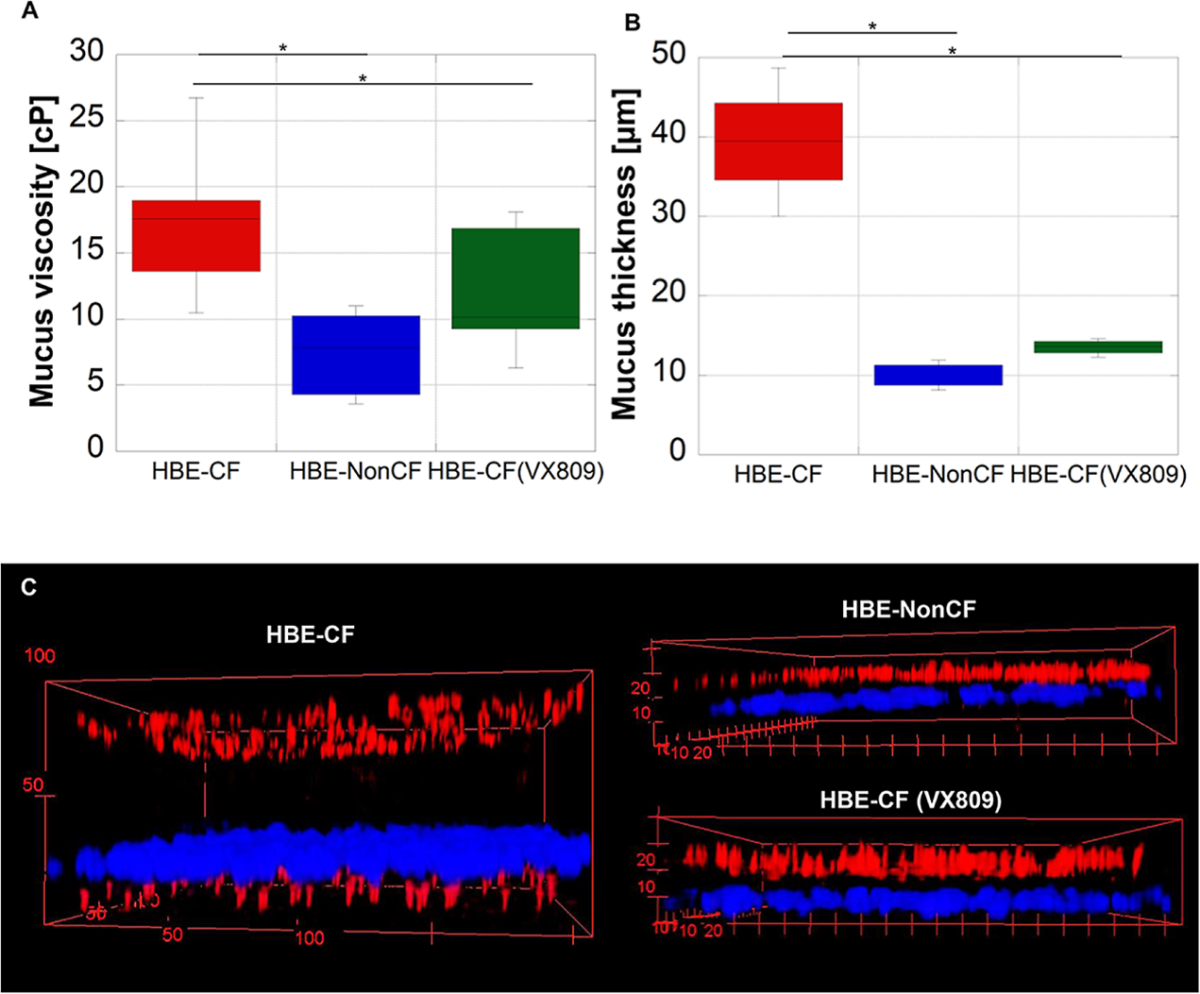
VX809 treatment on-chip: (A) box plot of mucus viscosity (cP) of bronchial epithelia differentiated on-chip (CF, CF after treatment with 3 μM VX809 for 48 h, and non-CF); (B) box plot of mucus thickness (μm) of bronchial epithelia differentiated on-chip (CF, CF after treatment with 3 μM VX809 for 48 h, and non-CF); and (C) 3D view showing red fluorescent nanoparticles (500 nm diameter) aerosolized on the surface of the sample to mark mucus thickness and epithelial nuclei marked in blue by Hoechst. *p value <0.05. The figure represents results from BE115 CF cells and BE122 nonCF for multiple particle tracking and BE86 CF and BE37 nonCF cells for the analysis of mucus thickness.

The reduced ciliary beat frequency (Figure 4H) was accompanied by a relevant lowering of the ensemble-averaged MSD curves of particles embedded in HBE-CF mucus in comparison with normal mucus. The MSD curve was higher in HBE-NonCF vs HBE-CF both in static and dynamic, but the culture condition on-chip enhanced this difference (Supporting Information S11A). Mucus viscosity calculated by using eqs 8 and 9 or eq 11 was significantly higher on the HBE-CF epithelium vs HBE-NonCF on-chip (Figure 5A), a well-known consequence of CFTR dysfunction in the airways.^40^ Indeed, the decreasing water release and the continuous mucus secretion are responsible for the hyper-concentration of the apical surface liquid and the increase of mucus viscosity in CF.^62^ At the same time, to evaluate mucus thickness, we placed an aerosol system on the sample previously stained by Hoechst to mark cell nuclei. Fluorescent nanoparticles were aerosolized on the apical surface of the sample to homogeneously mark the mucus without dilution. Supporting Information S12 shows the system used to aerosolize the fluorescent nanoparticle on the mucus surface (Supporting Information S12A) and a design of the principle (Supporting Information S12B). Aerosolization was performed on live samples for 30 s, the time necessary to homogeneously distribute drops with a diameter of 7–9 μm on the sample (Supporting Information S12C). Video S13 shows the aerosol system (Aerogen Solo) in function. After that, samples were observed by using a confocal microscope, and 3D view *z*-stack acquisitions allowed us to to evaluate mucus thickness as the empty space between the red particles up layer and the bottom layer of cells whose nuclei (blue) are marked with the vital nuclear dye Hoechst (Figure 5C). Results show that mucus thickness was higher in the HBE-CF epithelium vs HBE-NonCF on-chip (Figure 5B,C). Thus, the observed reduction of ciliary beat frequency was a consequence of the accumulation of dense and thick mucus, hindering cilia movement. The measure of mucus viscosity was lower than that in vivo, which is reported in the range of Pa s (1 Pa s = 1000 cP).^63^ Indeed, in vitro, the mucus is produced by a limited number of bronchial cells in culture, and it is directly collected by washing the epithelial surface with a saline solution and consequently diluting it. Instead, the mucus collected in vivo is produced by multiple cells of the airway and collected by bronchoalveolar lavage, sputum, bronchoscopy, or endotracheal tube sampling. These techniques allow for the retrieval of a large volume of mucus that can be analyzed directly by rheological test.^64^ Afterward, the chip was used for the administration of the corrector Lumacaftor (VX809) to HBE-CF epithelia. Indeed, the microfluidic chip was fabricated not only to culture and differentiate the CF bronchial epithelium but also to administer drugs through the basolateral flow in a time-controlled manner, thus mimicking the systemic administration of the drug in vivo. VX-809 is a CFTR corrector able to partially correct protein folding and allow CFTR insertion into the plasma membrane and function.^64^ Gianotti et al. demonstrated that the correction of F508del-CFTR mutation with lumacaftor was enough to improve significantly mucus properties.^40^ After 48 h of treatment with 3 μM VX-809 on-chip, we observed a slight increase of the ensemble-averaged MSD curves (Supporting Information S11B) and, therefore, a significant reduction of both mucus viscosity and thickness (Figure 5A–C). As a proof that mucus change was the result of CFTR correction, we analyzed mucus viscosity in control samples, obtained by adding just DMSO (that is, the diluent of the VX809) in the culture medium in the basolateral side. We observed that a low concentration of DMSO (<0.1% v/v) induced an increase of mucus viscosity in the apical side (from 18 ± 11 to 33 ± 11 cP) (Supporting Information S14). The increase of mucus viscosity in samples treated with DMSO was in line with experiments performed by Yuan et al.^65^ who reported an increase of the viscous modulus of porcine gastric mucin exposed to DMSO. In light of this, the reduction of mucus viscosity in presence of the VX809 acquires more significance, and it may be more evident if the VX809 was diluted in a different solvent.

We evaluated that the thickness of the mucus produced in the absence of the corrector was 39.4 ± 7.6 μm, similarly as reported for human bronchial epithelia in vivo,^43^ whereas the mucus produced in the presence of VX809 was 14.8 ± 3 μm in height, a value approaching the normal sample in which mucus thickness was 9.75 ± 1.7 μm.

To investigate the possibility to use the aerosol as a drug delivering tool, we performed a preliminary experiment by administrating VX809 via aerosol to the apical side of the epithelium on-chip. We observed a reduction of mucus viscosity in samples treated by VX809 vs DMSO delivered by aerosol (*p* value < 0.05). The viscosity of samples treated with VX809 was 21 ± 3 cPoise, whereas in control samples (DMSO), it was 33 ± 10 cPoise. We observed an increase of mucus viscosity in control samples (DMSO) (Supporting Information S15). However, this increase was in line with the literature^65^ and results obtained through the basolateral side.

## Conclusions

In this study, we showed the development of an easy-to-handle microfluidic chip for the culture, differentiation in a short time, and treatment of CF human bronchial epithelia. Tissues were grown at the ALI on-chip for 15 days. The chip allowed tissue oxygenation and feeding. The flow of the culture medium at the basal side of the sample generated shear stresses able to stimulate tissue differentiation by ciliary development and mucus secretion. Moreover, the epithelia on-chip acquired an elongated morphology. Impedance analysis of the tissue on-chip showed a TEER peak at day 3, lower TEER in the HBE-CF vs HBE-NonCF, and higher capacitance in the HBE-CCF vs HBE-NonCF. The dynamic condition highlighted differences between pathological and normal samples with lower ciliary beat frequency and higher mucus viscosity and thickness in HBE-CF. Tissue on-chip response to treatment with the corrector VX-809 was registered as a decrease in mucus viscosity and thickness, demonstrating the potential use of the microfluidic system to validate therapies administered through the basal side. In this respect, the fluidics could allow time-controlled administration of the drug, thus mimicking fluctuations in drug concentrations that occur in vivo due to drug pharmacokinetic characteristics. Moreover, in this study, we used an aerosol system to deliver fluorescent nanoparticles on the apical side of the sample and detect mucus thickness. We expect that the same tool may be used to deliver drugs on the surface of the sample to mimic aerosol administration in vivo. Therefore, we argue that the proposed CF epithelium on-chip may be a tool useful for the study of CF and drug testing, in a more physiologically dynamic environment. As a future perspective, the dynamic co-culture of human bronchial epithelial cells with lung fibroblasts and endothelial cells into a native ECM would significantly increase the complexity of the system and its proximity to the human tissue.

## Supporting Information Available

Aditional experimental details, materials, and methods, including photographs and videos of experimental setup on-chip; detailed list of the supplementary materials: S1 microfluidic device, S2 transwell insertion on-chip (VIDEO), S3 setting for dynamic culture (VIDEO), S4 microfluidic setup, S5 epithelial differentiation on-chip at heterogeneous shear stress, S6 LDH activity assay, S7 setting for electrical measurements, S8 table of TEER values, S9 electrical and morphological properties of the CF-HBE over time, S10 Forskolin assay and capacitance measurement, S11 MDS plots, S12 aerosol system to detect mucus thickness, S13 aerosol system (VIDEO), S14 analysis of mucus viscosity of CF samples after treatment from the basal side of the insert, S15 analysis of mucus viscosity of CF samples after treatment from the apical side of the insert (ZIP)
Microfluidic device, microfluidic setup, epithelial differentiation on-chip at heterogeneous shear stress, LDH activity assay, setting for electrical measurements, TEER values, electrical and morphological properties of the CF-HBE over time, forskolin assay and capacitance measurement, MDS plots, aerosol system to detect mucus thickness, and analysis of mucus viscosity of CF samples (PDF)
