# Supplementary material for: Easy-to-Build and Reusable Microfluidic Device for the Dynamic Culture of Human Bronchial Cystic Fibrosis Epithelia
Source: ACS Biomater Sci Eng. 2023 Apr 5;9(5):2780–92. doi: 10.1021/acsbiomaterials.2c01460 (PMC10170479; doi:10.1021/acsbiomaterials.2c01460)
Supplement: Supplementary file 2 — ab2c01460_si_002.pdf [file ab2c01460_si_002.pdf]

An easy-to-build and re-usable microfluidic device for the dynamic culture of human bronchial cystic fibrosis epithelia

*Claudia Mazio[1, #], Laura S. Scognamiglio[1, #], Roberta Passariello[1,2], Valeria Panzetta[2,3], Costantino Casale[3], Francesco Urciuolo[2,3], Luis J. V. Galietta[4], Giorgia Imparato[1, *] and Paolo A. Netti[1,2,3, *]*

[1] Istituto Italiano di Tecnologia (IIT) - Center for Advanced Biomaterials for Healthcare, Largo Barsanti e Matteucci 53, 80125, Napoli, Italy

[2] Department of Chemical, Materials and Industrial Production Engineering (DICMAPI) University of Naples Federico II, P.le Tecchio 80, 80125 Naples, Italy

[3] Interdisciplinary Research Centre on Biomaterials (CRIB), University of Napoli Federico II, P.le Tecchio 80, 80125, Napoli, Italy

[4] Telethon Institute of Genetics and Medicine (TIGEM), Via Campi Flegrei 34, 80078, Pozzuoli (NA), Italy

[#] These authors equally contributed to the manuscript

[*] Corresponding authors: giorgia.imparato@iit.it; paolo.netti@iit.it

SUPPORTING INFORMATION

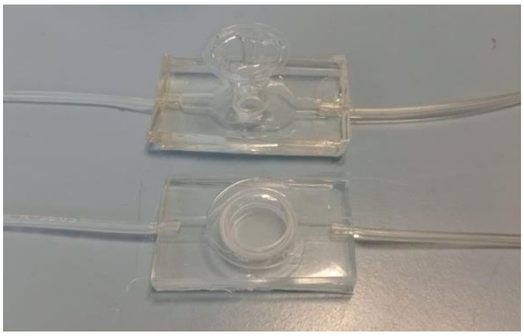

S 1 Microfluidic device: Representative pictures of the microfluidic chip with a Transwell (up) or a Snapwell (down) insert for cell culture

S2 Transwell insertion on-chip (VIDEO): The video shows the simplicity of manual insertion of the transwell on-chip and the stability of the integrated insert.

S3 Setting for dynamic culture (VIDEO): The video shows the dynamic setting comprising the microfluidic device attached to a reservoir by tubing and connectors and the establishment of the dynamic flow circuit through the use of a peristaltic pump.

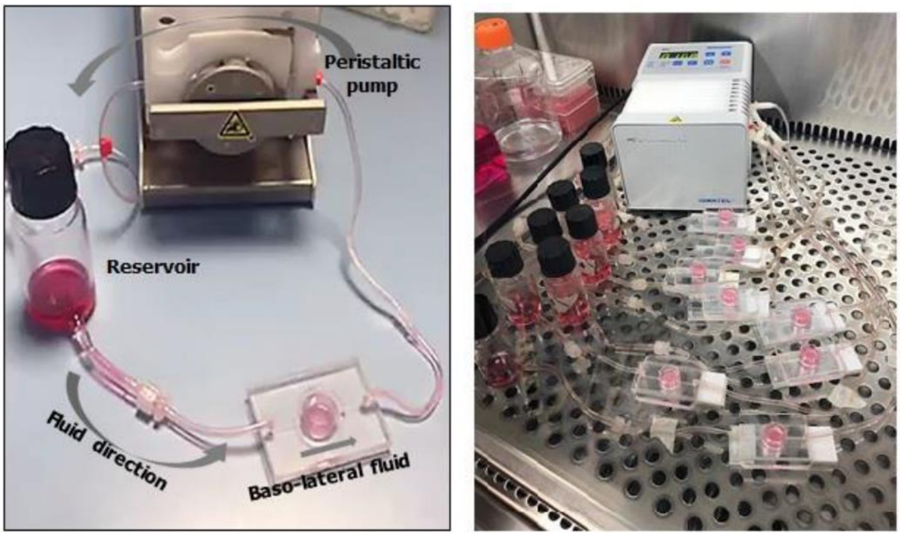

S 4 Microfluidic setup: pictures of the microfluidic device for a single (left) or multiple devices in parallel (right).

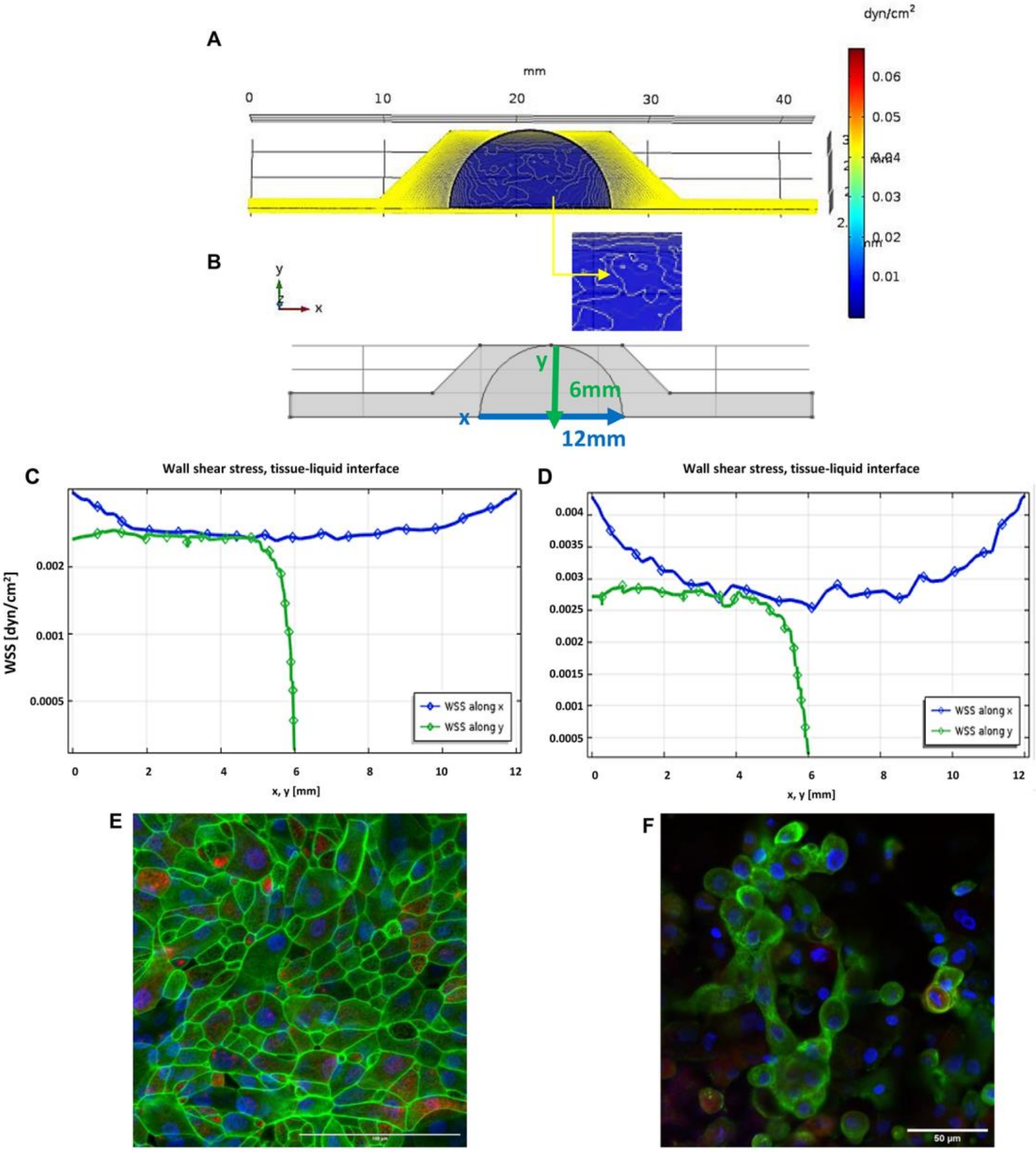

S 5 Epithelial differentiation on-chip at heterogeneous shear stress: A) Flux lines distribution on the chip with smaller channels and colormap of shear stresses; B)Design of the microfluidic chip with the x and y directions highlighted by the light blue and green arrows, respectively; B) Shear stress distributions in the x (light blue) and y (green) directions of the microfluidic chip with larger channels (15x4x1.5 mm); C) Shear stress distributions in the x (light blue) and y (green) directions of the microfluidic chip with smaller channels (15x1x1.5 mm); E) Immunofluorescence for Alpha Tubulin in red, Actin in green (marked by Phalloidin), cell nuclei in blue (marked by DAPI) in the chip with larger channels; F) Immunofluorescence for Alpha Tubulin in red, Actin in green (marked by Phalloidin), cell nuclei in blue (marked by DAPI) in the chip with larger channels. Sample cultured in dynamic by using a low flow rate of the peristaltic pump (Q=40µl/min); B) Scale Bar 50µm; C) Scale bar 100 µm.

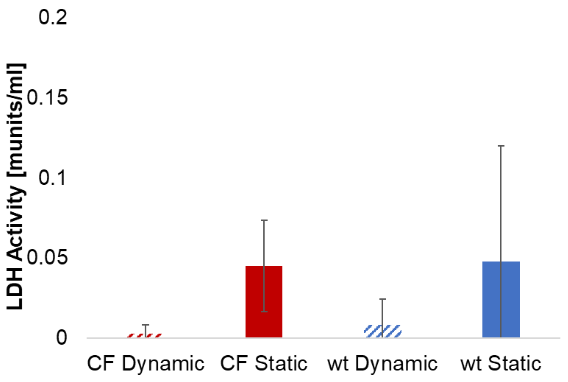

S 6 LDH Activity assay: The bar plot shows the LDH activity (munits/ml) in normal and cystic fibrosis epithelia both in dynamic and static, after 14 days of culture. Graphs report mean values ± SD.

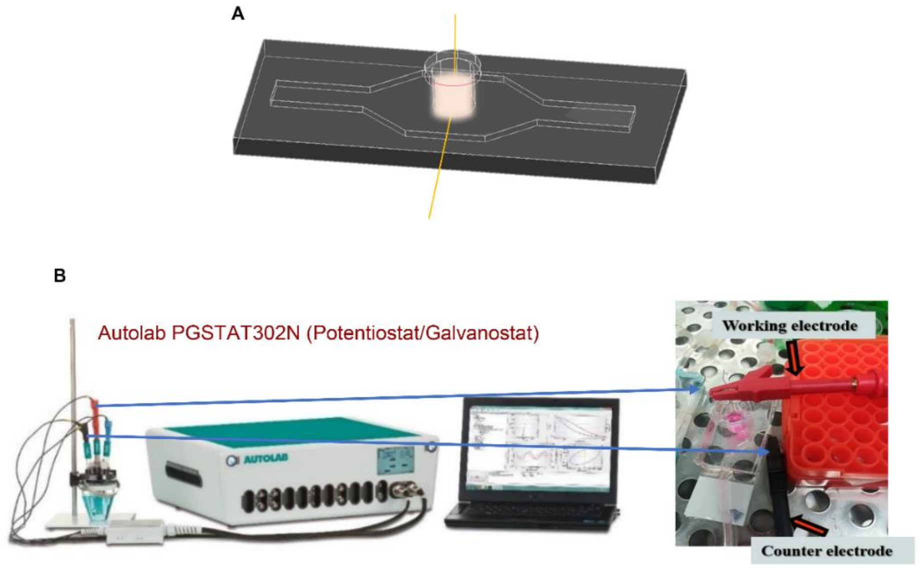

S 7 Setting for electrical measurements: A) Microfluidic device with two gold electrodes for impedance analyses, the electrode on the bottom was integrated into the device meanwhile the second was added directly for the measures; B) The electrode on the top was mounted on a base (orange) and connected to the working electrode of the Potentiostat, the electrode on the bottom was connected to the counter electrode.

	HBE-CF	HBE-NonCF
**TEER (Ω) dynamic**	1409.429± 585.1264	1588± 457.0631
**TEER (Ω) static**	322.2± 153.5005	326.625± 147.6404295

S 8 Table of TEER values: The table reports the Mean TEER values ± SD of HBE-CF and HBE-NonCF in dynamic and static at day 3.

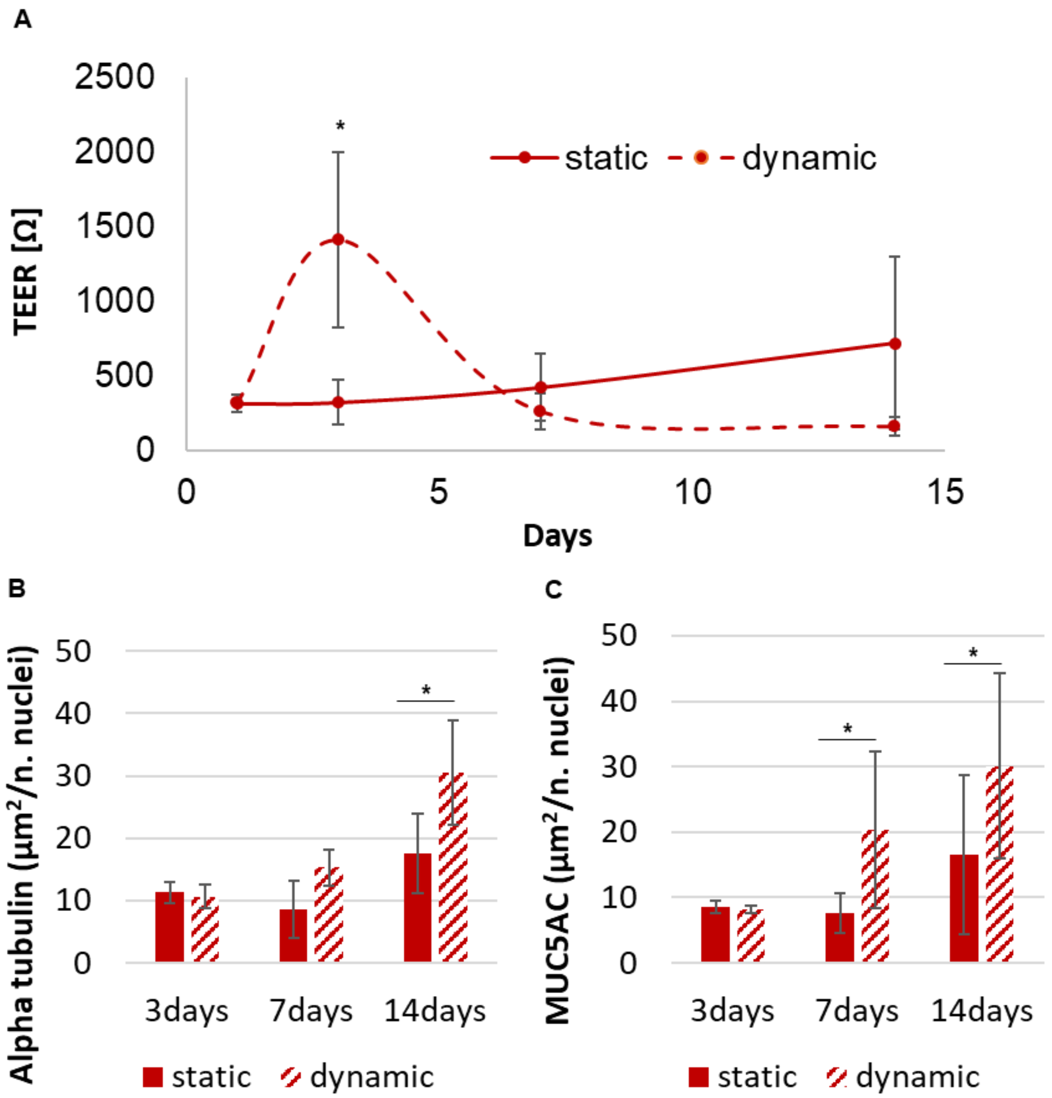

S 9 Electrical and morphological properties of the CF-HBE over time: A) Trend of Rteer measures for CF-HBE in dynamic and static conditions; B) Quantification of the Alpha Tubulin signal (μm2 / number of nuclei) in static vs dynamic over time culture; C) Quantification of the MUC5AC signal (μm2 / number of nuclei) in static vs dynamic over time culture; *p value < 0.05.

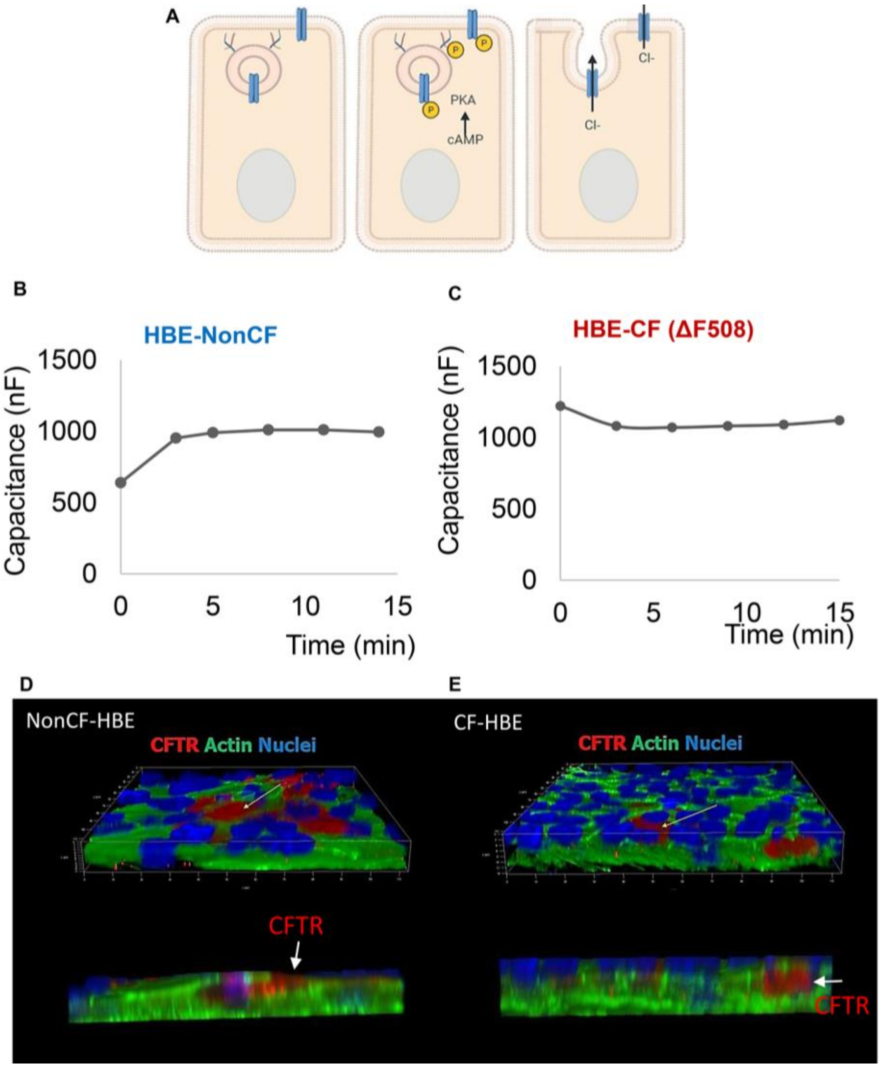

S 10 Forskolin assay and capacitance measurement: A) Design showing the integration of a vesicle carrying CFTR into the plasma membrane. cAMP activation by forskolin can stimulate the PKA-dependent exocytosis of CFTR-containing vesicles and their insertion into the plasma membrane with consequent increase of the membrane length; image created with BioRender.com; B) Capacitance measure (nF) of non-cystic fibrosis human bronchial epithelia (HBE-NonCF) after forskolin stimulation, C) Capacitance measure (nF) of cystic fibrosis human bronchial epithelia (HBE-CF) after forskolin stimulation; D) 3D view showing CFTR in red, cell actin in green and cell nuclei in blue, in normal epithelia the ~~green~~ arrow highlights the presence of apical CTRF after Forskolin induction; the transversal section of the sample is reported on the bottom to highlight CFTR apical localization E) 3D view showing CFTR in red, cell actin in green and cell nuclei in blue, in cystic fibrosis epithelia the green arrow highlights the presence of cytoplasmic CTRF (because it is localized below the cell nucleus) after Forskolin induction; the transversal section of the sample is reported on the bottom to highlight CFTR sub-apical localization. The graphics in B and C display capacitance change after forskolin stimulation. Time 0 is before the addiction of Forskolin in the apical side of the sample. Afterward, forskolin was added and the capacitance was continuously monitored every 3 minutes for 15 minutes.

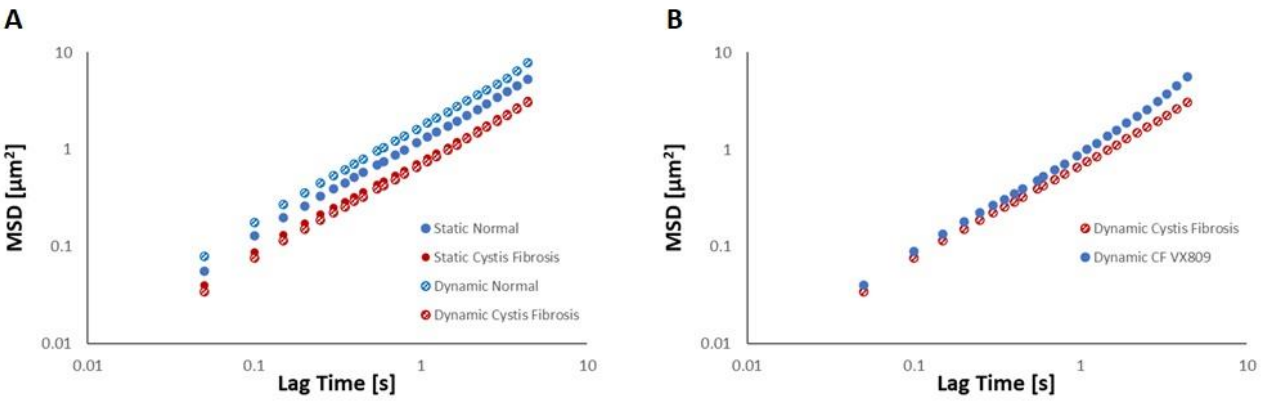

S 11 MDS Plots: The MSDs of 200 nm nanoparticles in mucus produced by normal (blue circles) and cystic (red circles) epithelia in static (solid circles) and dynamic (textured circles) conditions. In both static and dynamic conditions, the ensemble-averaged MSD curve of particles **embedded** in CF mucus is lower than in normal mucus. A) MSDs in the static and dynamic condition of normal and cystic fibrosis samples; B) Comparison of particle MSDs in dynamic cystic fibrosis samples treated (blue) or not (red).

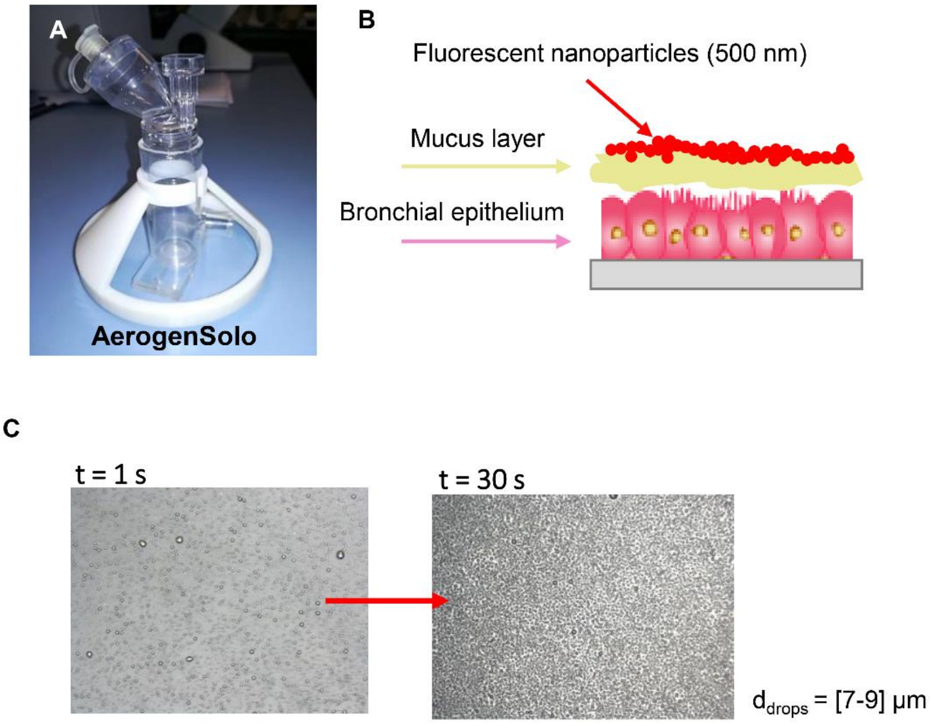

S 12 Aerosol system to detect mucus thickness: A) Picture of the Aerosol system; B) Design showing the delivery of red fluorescent nanoparticles and their accumulation on the apical mucus; C) Brightfield images of the drops released by the aerosol on a transparent surface after 1 and 30 seconds, drops with a diameter of about 7-9µm covered the surface after 30 seconds.

S13 Aerosol system (VIDEO): The video shows the aerosol system (Aerogen Solo) in function. The aerosol was attached to a glass column with a lateral opening as solid support placed on the device.

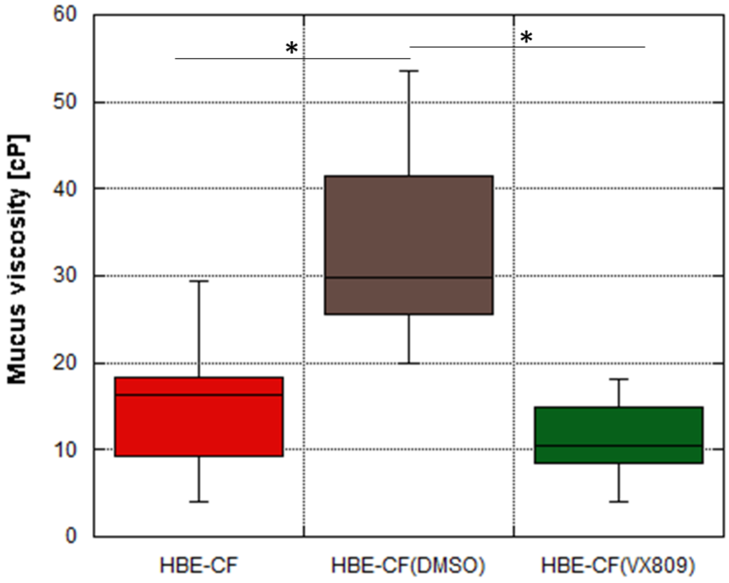

S 14 Analysis of mucus viscosity of CF samples after treatment from the basal side of the insert: Box plot of mucus viscosity (cP) of cystic fibrosis bronchial epithelia differentiated on-chip: HBE-CF are non-treated samples, HBE-CF (DMSO) are negative controls; HBE-CF (VX809) are samples treated with VX809 from the basolateral side of the insert; *p value < 0.05.

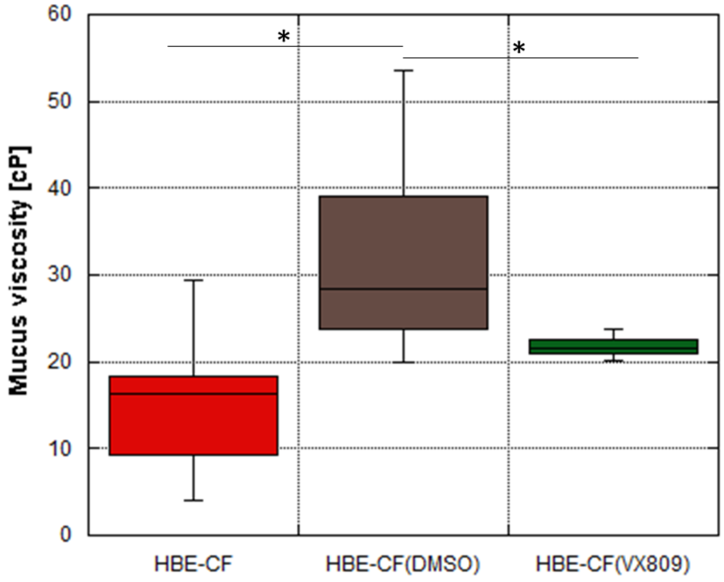

S 15 **Analysis of mucus viscosity of CF samples after treatment from the apical side of the insert:** Box plot of mucus viscosity (cP) of cystic fibrosis bronchial epithelia differentiated on-chip: HBE-CF are non-treated samples, HBE-CF (DMSO) are negative controls; HBE-CF (VX809) are samples treated with VX809 by aerosol, from the apical side of the insert; *p value < 0.05.
